# The organizing role of Wnt signaling pathway during arthropod posterior growth

**DOI:** 10.3389/fcell.2022.944673

**Published:** 2022-08-05

**Authors:** Marco Mundaca-Escobar, Rodrigo E. Cepeda, Andres F. Sarrazin

**Affiliations:** CoDe-Lab, Instituto de Química, Pontificia Universidad Católica de Valparaíso, Valparaíso, Chile

**Keywords:** signaling pathway, segment addition zone, body segmentation, axial elongation, wnt signaling

## Abstract

Wnt signaling pathways are recognized for having major roles in tissue patterning and cell proliferation. In the last years, remarkable progress has been made in elucidating the molecular and cellular mechanisms that underlie sequential segmentation and axial elongation in various arthropods, and the canonical Wnt pathway has emerged as an essential factor in these processes. Here we review, with a comparative perspective, the current evidence concerning the participation of this pathway during posterior growth, its degree of conservation among the different subphyla within Arthropoda and its relationship with the rest of the gene regulatory network involved. Furthermore, we discuss how this signaling pathway could regulate segmentation to establish this repetitive pattern and, at the same time, probably modulate different cellular processes precisely coupled to axial elongation. Based on the information collected, we suggest that this pathway plays an organizing role in the formation of the body segments through the regulation of the dynamic expression of segmentation genes, *via* controlling the *caudal* gene, at the posterior region of the embryo/larva, that is necessary for the correct sequential formation of body segments in most arthropods and possibly in their common segmented ancestor. On the other hand, there is insufficient evidence to link this pathway to axial elongation by controlling its main cellular processes, such as convergent extension and cell proliferation. However, conclusions are premature until more studies incorporating diverse arthropods are carried out.

## Introduction

Panarthropods are a superphylum integrated by organisms with bilateral symmetry and a segmented body, divided into Tardigrada, Onychophora, and Arthropoda. The latter has been the most studied so far and is, in turn, grouped into Chelicerata (*e.g.*, spiders and scorpions), Myriapoda (*e.g.*, centipedes and millipedes), and Pancrustacea (*e.g.*, crustaceans and insects) (Giribet and Edgecombe, 2019). The embryonic development of panarthropods is based on the organization of a segmented body plan, which is a shared feature in their evolutionary history (Hannibal and Patel, 2013; Chipman and Edgecombe, 2019). This developmental modality is based on the formation of similar repetitive units called segments along the anteroposterior axis (Clark et al., 2019). The establishment of this segmented patterning has been considered a significant contribution to promoting the great diversity of shapes and sizes found within panarthropods and their high adaptive success in practically all the environments on our planet (Peel et al., 2005; Auman and Chipman, 2017).

The segmented body plan of panarthropods is orchestrated by a wide variety of cellular mechanisms and genetic regulators, which can vary depending on the organism in question.

A widely conserved signaling pathway during embryonic development is the Wnt pathway, which coordinates crucial cellular processes such as proliferation, cell polarity, and the determination of cell fate (Williams and Nagy, 2017; Steinhart and Angers, 2018). Three main different Wnt signaling pathways have been described: The canonical pathway, which is dependent on *β*-catenin cytoplasm accumulation and subsequent nuclear translocation to the coactivation of specific gene transcription, and the noncanonical Wnt/planar cell polarity (Wnt/PCP) and Wnt/Ca^2+^ pathways, which mediate cytoskeleton dynamics and cell movements through directional information or intracellular Ca^2+^ release, respectively (Croce and McClay, 2008; Angers and Moon, 2009). Although canonical and noncanonical pathways have different signaling mechanisms, both participate in regulating different cellular processes and genetic patterning, allowing successful embryonic development.

The first studies of the Wnt pathway in panarthropods were carried out on the vinegar fly *Drosophila melanogaster* (Nüsslein-Volhard and Wieschaus, 1980; Baker, 1987; Rijsewijk et al., 1987; Martinez et al., 1988), initiating a wide line of research that linked this pathway to embryonic development, revealing its relationship with various developmental processes. Over the years, more research has been conducted on flies and several vertebrates, including diverse developmental processes and diseases (Jenny and Basler, 2014; Bejsovec, 2018; Wiese et al., 2018), but neglecting the high number of existing arthropods and their different modes of embryogenesis (Murat et al., 2010).

In the last years, exciting advances have been made regarding the molecular and cellular mechanisms involved during sequential segmentation and axial elongation in a variety of arthropods, and the Wnt pathway has emerged as an essential factor in these processes. Some reviews have covered this topic while not focusing specifically on the role of this signaling pathway (Martin and Kimelman, 2009; Williams and Nagy, 2017; Clark et al., 2019). Thus, in a comparative approach, it remains to discuss how and when the Wnt pathway participates in establishing the segmented pattern and regulating processes such as cell proliferation or convergent extension during the posterior body growth in panarthropods. In this review, we will start with a description of the repertoire and expression pattern of Wnt ligands in a vast number of panarthropods, including representatives of all the phyla, addressing their possible roles during germband extension. Then, we will discuss how this signaling pathway could regulate segmentation to establish this repetitive pattern and, at the same time, probably modulate different cellular processes precisely coupled to axial elongation.

## The repertoire of Wnt ligands present in panarthropods

The Wnt pathway is a complex signaling pathway present in all metazoans–including placozoans and sponges–that comprises several receptors and intracellular components, as well as an established repertoire of thirteen ligand subfamilies (Holstein, 2012). In vertebrates, it has been determined that Wnt1, Wnt2, Wnt3, Wnt8, and Wnt10 ligands are activators of the canonical pathway, and that Wnt4, Wnt5, Wnt6, Wnt7, Wnt9, and Wnt11 ligands are activators attributed to the noncanonical pathways (Gajos-Michniewicz and Czyz, 2020), while Wnt16 ligand has been shown to be an activator of both, canonical and noncanonical pathways (Gori et al., 2015). WntA ligand, of an indeterminate group, has not been found in vertebrates (Prud’homme et al., 2002), although it is present in urochordates and cephalochordates (Somorjai et al., 2018), as well as in panarthropods (Janssen et al., 2010; Hogvall et al., 2014).

**FIGURE 1:**
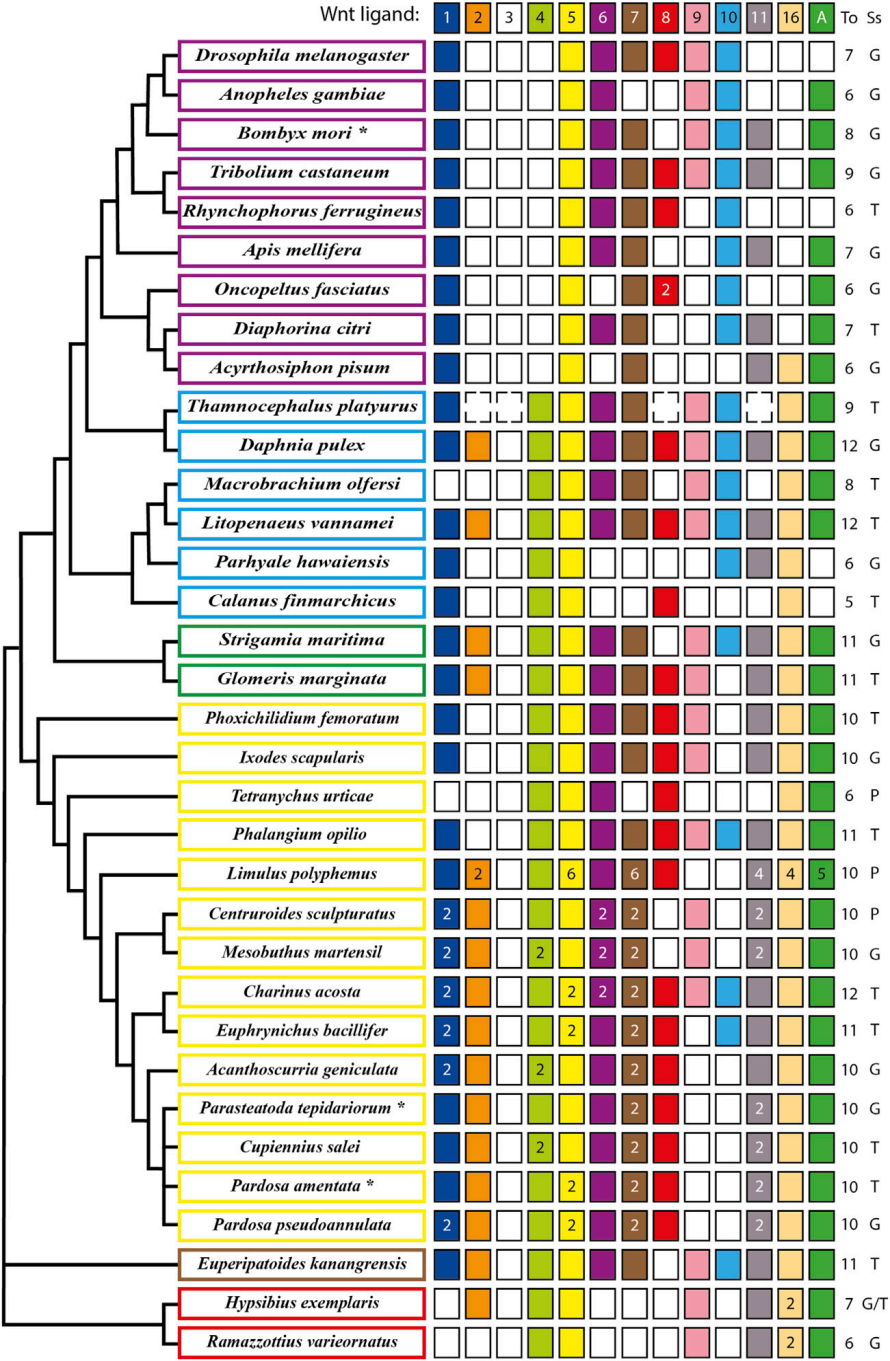
Updated repertoire and phylogenetic relationship of Wnt ligands among panarthropods. Insects are shown in purple rectangles (Dearden et al., 2006; Bolognesi et al., 2008a; Murat et al., 2010; Shigenobu et al., 2010; Yin et al., 2015; Ding et al., 2019; Holzem et al., 2019; Panfilio et al., 2019; Vosburg et al., 2020), crustaceans in pale blue (Janssen et al., 2010; Constantinou et al., 2016; Jaramillo et al., 2016; Kao et al., 2016; Du et al., 2018), myriapods in green (Janssen et al., 2010; Hayden and Arthur, 2014; Janssen and Posnien, 2014), chelicerates in yellow (Janssen et al., 2010; Pace et al., 2014; Posnien et al., 2014; Harper et al., 2021; Janssen et al., 2021; Janssen and Eriksson, 2022), onychophoran in brown (Hogvall et al., 2014) and tardigrades in red (Chavarria et al., 2021). An asterisk in the name indicates more organisms having the same repertoire. *B. mori also includes *Bicyclus anynana*, *Amyelois transitella*, *Calycopis cecrops*, *Danaus plexippus*, *Heliconius melpomene*, *Operophtera brumata* and *Papilio xuthus*. *P. tepidariorum includes *Pholcus phalangioides* and *P. amentata includes *Marpissa muscosa* and *Stegodyphus dumicola*. Filled and no-filled boxes represent the presence and the absence of the ligand, respectively. Dotted boxes represent the presence/absence of the ligand in doubt (genome not fully sequenced) and white/black numbers inside the boxes represent ligand numbers. The Wnt ligand family is indicated at the top as well as the total (To) number of ligand families. The source of the sequences (Ss) is showed as Genome (G), Transcriptome (T) or Proteome (P). Phylogenetic positions based on Koenemann et al., 2010, Misof et al., 2014, Giribet and Edgecombe, 2019, Lozano-Fernandez et al., 2019 and Ballesteros et al., 2022.

The analysis of the full repertoire of Wnt ligands in panarthropods published so far (Figure 1) showed that a common characteristic is the loss of the Wnt3 ligand, implying that their common ancestor most probably presented ligands from only twelve subfamilies (Hayden and Arthur, 2014; Hogvall et al., 2014; Janssen and Posnien, 2014). A significant loss also occurred in insects, where the absence of Wnt2 and Wnt4 ligands in all the representatives is observed. Interestingly, an ortholog of *wnt16* is present in the apid and absent in the other eight insects included in the analysis (Dearden et al., 2006; Bolognesi et al., 2008a; Murat et al., 2010; Shigenobu et al., 2010; Yin et al., 2015; Ding et al., 2019; Holzem et al., 2019; Panfilio et al., 2019; Vosburg et al., 2020). More losses are found in other lineages. However, they were not general to the clade, as they are mixed with other species that retained at least ten Wnt ligands from the ancestral twelve subfamilies (Figure 1). While these losses may depict specific eliminations in particular species within Arthropoda, we cannot exclude that missing ligands could represent low expression transcripts rather than losses at the genomic level when the database used is a transcriptome instead of the whole sequenced genome, as in the cases of *Cupiennius salei*, *Calanus finmarchicus* and *Thamnocephalus platyurus*, among others.

Our updated repertoire also showed that only Wnt4, Wnt5 and Wnt16 ligands were retained in all crustacean lineages–represented here by six species that belong to three of the six extant classes (Zhang, 2011) –, with a low degree of conservation of the Wnt2, Wnt8, and Wnt11 ligands (Constantinou et al., 2016; Jaramillo et al., 2016; Kao et al., 2016; Du et al., 2018).

Regarding chelicerates, they present a low loss of ligands. They also exhibit a high degree of duplications in almost all Wnt ligand subfamilies, which coincides with the whole genome duplication (WGD) that has been proposed to have taken place in the common ancestor of spiders and scorpions (Arachnopulmonata) (Schwager et al., 2017; Panfilio et al., 2019; Harper et al., 2021). Furthermore, an extreme case is found in the horseshoe crab *Limulus polyphemus*, that exhibit up to six copies of Wnt5 and Wnt7 ligands, most probably due to several rounds of WGD that have been recently documented for this order - Xiphosura - of chelicerates (Nossa et al., 2014; Nong et al., 2021). This leaves out harvestmen, sea spiders, ticks, and mites, represented in our compilation by *Phalangium opilio*, *Phoxichilidium femoratum*, *Ixodes scapularis*, and *Tetranychus urticae*, respectively, which show no duplications (Figure 1). Interestingly, *T. urticae* and the decapod crustacean *Macrobrachium olfersi* both lacks one of the most conserved ligands, Wnt1—only Wnt5 is fully present among panarthropods–and only retained one canonical ligand, Wnt8, and Wnt10, respectively. Thus, despite multiple Wnt ligand losses, there is always a canonical/noncanonical set of ligands in all reported species; however this repertoire is more variable for some ligands than for others. This diversity and variability within arthropods have been explained before by non-conserved specific functional redundancies among Wnt ligands in this and other phyla that are evident after single ligand loss-of-function experiments where no strong defects are found in or related to the site of expression (*e.g.*, Gleason et al., 2006; Bolognesi et al., 2008a; Grigoryan et al., 2008).

Additionally, there are multiple lines of evidence of Wnt ligands signaling by canonical and noncanonical pathways. For example, *Drosophila wnt9* (*Dwnt4*) activates cell motility during ovarian morphogenesis through the noncanonical pathway and salivary gland development through the canonical pathway (Cohen et al., 2002; Harris et al., 2007). In another example, human isolated chondrocytes differentiation was triggered *in vitro* by *wnt3a* that simultaneously activated both Wnt/β-catenin and Wnt/Ca^2+^ signaling pathways (Nalesso et al., 2011). All this makes the interchangeability between different ligands or their combinatorial activity to regulate the same pathways or trigger similar functions even more feasible.

On the other hand, *wnt* genes duplication found almost exclusively in chelicerates has allowed this group to reach up to 14–16 ligands, keeping paralogs of the same Wnt ligand subfamily despite having several other ligands. Some authors have attributed this to an ancestral division of the function between both paralogs and acquisition of a new function by one of them (Gitelman, 2009; Cho et al., 2010). It has also been suggested that larger sets of ligands could be an indication of morphological and functional diversification (Schwager et al., 2017). However, since numerous Wnt ligand subfamilies were found in simpler and basally branching metazoans, for example twelve in the sea anemone *Nematostella vectensis*, ten in the demosponge *Halisarca dujardini* and even 21 in the calcisponge *Sycon ciliatum*, there is no obvious relationship between higher number of ligands and morphological complexity (Kusserow et al., 2005; Borisenko et al., 2016).

## The expression domains of Wnt ligands

To fully understand the wide and varied range of Wnt ligands present in the different panarthropods studied so far, it is necessary to analyze their expression patterns in a comparative manner throughout development. In this review we will focus on the period comprising the processes of axial elongation and segmentation, collectively known as posterior growth.

**FIGURE 2:**
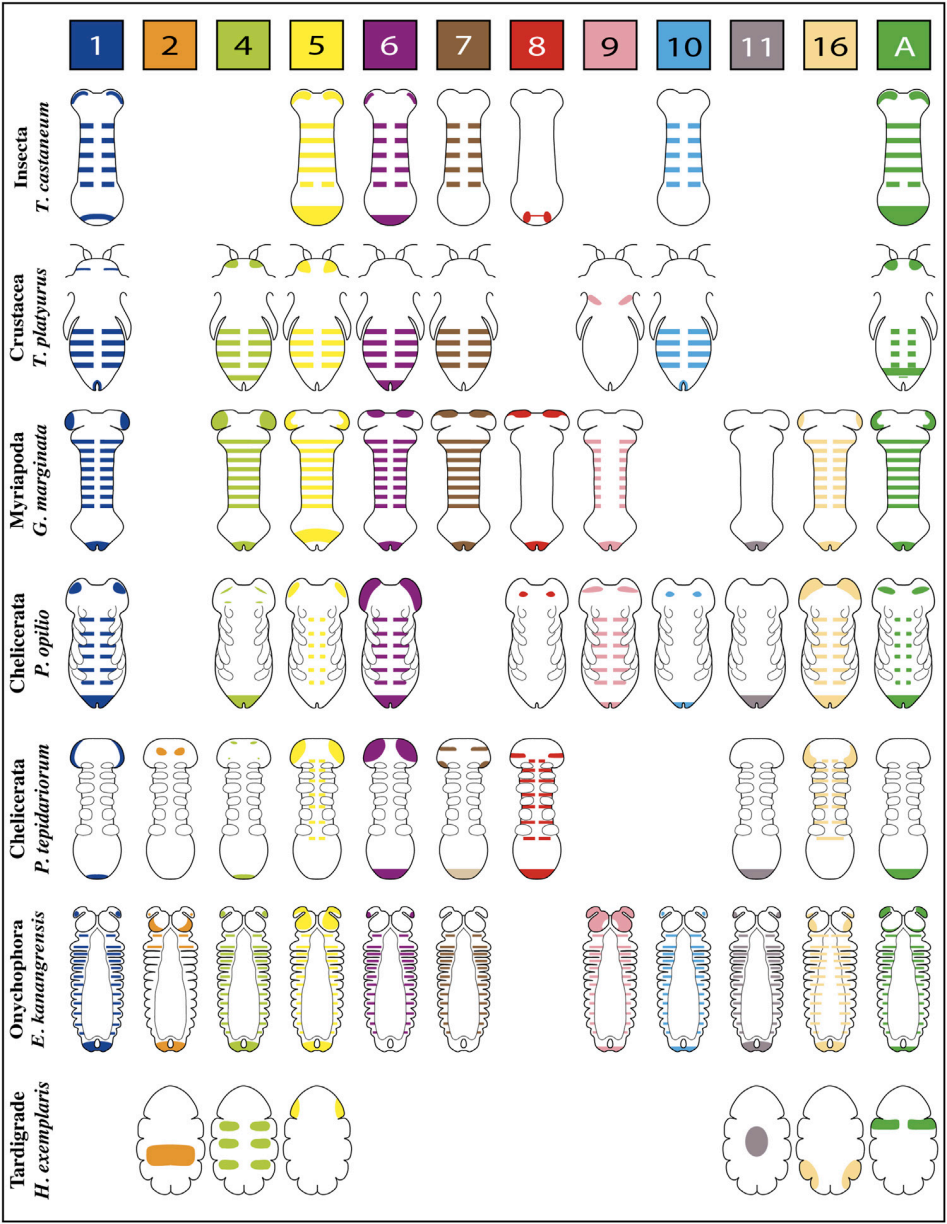
Representative expression patterns of Wnt ligands in panarthropods. Expression patterns of Wnt ligands in representative organisms of Insecta, Crustacea, Myriapoda, Chelicerata, Onychophora and Tardigrada. Each color represents one Wnt ligand as is indicated at the top. The Wnt7 ligand in *P. tepidariorum* presents duplication, therefore double expression is shown in the scheme (Wnt7.1 is pale brown in the posterior, Wnt7.2 is dark brown in the head). *T. castaneum* (Bolognesi et al., 2008a); *T. platyurus* (Constantinou et al., 2016); *G. marginata* (Janssen et al., 2010; Janssen and Posnien, 2014); *P. opilio* (Janssen et al., 2021); *P. tepidariorum* (Janssen et al., 2010; Janssen et al., 2021); *E. kanangrensis* (Hogvall et al., 2014); *H. exemplaris* (Chavarria et al., 2021).

**FIGURE 3:**
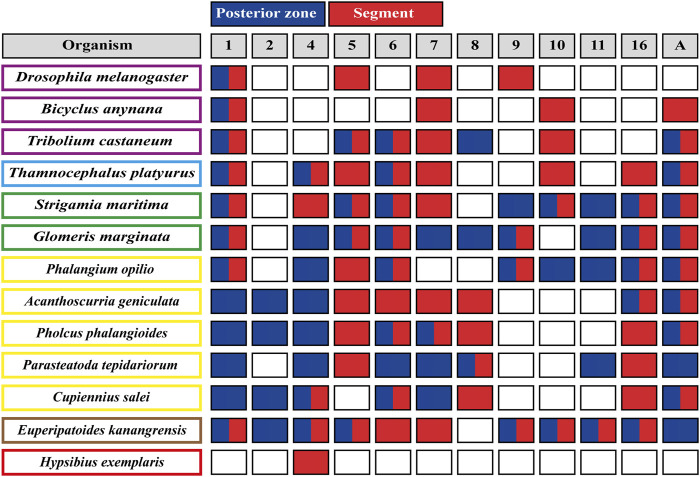
Wnt ligands expression domains within panarthropod germbands. Blues boxes represent the expression of the corresponding Wnt ligand in the posterior zone, red boxes indicate segmental expression, and white boxes show the absence of expression. Double red and blue boxes indicate Wnt ligand expression at the posterior zone and within segments. Insects are shown in purple rectangles, crustaceans in pale blue, myriapods in green, chelicerates in yellow, onychophoran in brown and tardigrades in red. The Wnt ligand families are indicated at the top, in gray boxes. *D. melanogaster* (Murat et al., 2010); *B. anynana* (Holzem et al., 2019); *T. castaneum* (Bolognesi et al., 2008a); *T. platyurus* (Constantinou et al., 2016); *S. marítima* (Hayden and Arthur, 2014); *G. marginata* (Janssen et al., 2010; Janssen and Posnien, 2014); chelicerates (Janssen et al., 2021; Janssen and Eriksson, 2022); *E. kanangrensis* (Hogvall et al., 2014); *H. exemplaris* (Chavarria et al., 2021).

Published expression patterns of the complete repertoire of Wnt ligands are relatively scarce in panarthropods. However, we found such data for three representative insect species–unfortunately not including hemimetabolous insects–one branchiopod crustacean, two myriapods–a centipede and a millipede,—four chelicerates–including only arachnids: three spiders and one harvestman–and one representative for onychophorans and one for tardigrades (Figures 2,3).

The spatiotemporal analysis of *wnt* genes expression in the beetle *Tribolium castaneum* showed that several ligands overlap in the posterior zone, segments/parasegments, and head lobes, suggesting functional redundancy (Bolognesi et al., 2008a). However, when we compiled *wnt* genes expression patterns including the other panarthropods (Janssen et al., 2010; Hogvall et al., 2014; Janssen and Posnien, 2014; Constantinou et al., 2016; Holzem et al., 2019; Chavarria et al., 2021; Janssen et al., 2021), we found that overlapping is common, although diverse patterns are still observed (Figure 2). Based on the degree of patterning conservation, we were able to classify the expression of Wnt ligands as highly (Wnt1, Wnt5, Wnt6, Wnt7, and Wnt11), moderately (Wnt8, Wnt10, Wnt16, and WntA), and poorly (Wnt2, Wnt4, and Wnt9) conserved, which might suggest that the function of some of these ligands–and not others–would be essential for posterior growth in most panarthropods. Furthermore, early-branching clades, such as onychophorans–a sister group of arthropods –, chelicerates, and myriapods, showed a higher proportion of Wnt ligands in the posterior zone compared to pancrustaceans (Figure 3). Despite all this, it is unclear whether overlapping ligands fulfill similar/redundant or different/complementary functions.

*wnt1* is expressed in the posterior region–known as the growth zone or, more precisely, the segment addition zone (SAZ)—of practically all the panarthropods analyzed except for the tardigrades, which do not have a proper SAZ (Chavarria et al., 2021). This suggests a leading and ancestral role of Wnt1 ligand in the regulation of the posterior growth in Panarthropoda.

Within arachnids, two *wnt1* paralogs in the tarantula *Acanthoscurria geniculata* show a clear example of posterior subfunctionalization, where one of them is only expressed on the SAZ while the other is expressed exclusively in the hindgut primordium. Interestingly, in arachnids which have retained only one paralog, *wnt1* is expressed at later stages in the putative hindgut of *Parasteatoda*–without expression in the SAZ–and in the SAZ of *Pholcus* and *Phalangium*, with no expression in the hindgut primordium (Janssen et al., 2021).

Specialized functions for *wnt1* and *wnt5* are suggested by their expression patterns within the posterior region of the *Tribolium* germband (Nagy and Carroll 1994; Bolognesi et al., 2008a). *wnt1* expression is restricted to a specific domain within the SAZ–resembling the *wnt8 domain*–in a region apparently covered by the broader expression of *wnt5*. Since both expression domains are not equivalents, they could be covering different functions.

An analysis of Wnt1 and Wnt5 ligands performed in the spider *Cupiennius salei* showed their complementary expression domains within the segment, dorsal for *wnt1* and ventral for *wnt5* (Damen, 2002). However, the suggested “complementarity” in *Cupiennius* was refuted by Janssen et al. (2021) arguing that *wnt5* ventral expression is restricted to the nervous system and not related to the segment polarity establishment.

On the other hand, in the onychophoran *Euperipatoides kanangrensis*, Hogvall et al. (2014) observed that distinct Wnt ligands were expressed at different and specific regions throughout each trunk segment; *wnt5* and *wnt4* extending over the anterior and posterior domains, respectively, while *wnt1*, *wnt6*, *wnt9*, *wnt10*, *wnt11*, and *wnt16* covering the center of the segment in adjacent or overlapping regions. However, these onychophoran *wnt* genes are expressed in a segment polarity-like pattern only at later stages, belatedly to segment polarity genes such as *engrailed*. Thus, the authors suggested that *wnt* genes were not involved in forming segmental borders in onychophorans but probably in their intrasegmental patterning.

In summary, a complete *wnt* genes repertoire is published and available in twelve panarthropods, including representatives of the main subphyla. Furthermore, many *wnt* genes are expressed in the forming segments and the posterior region during panarthropod elongation, with a general high overlapping among them. However, there is little detailed information on these expressions. Until a better resolution in the expression of various arthropods is available, both at the tissue and cellular level, it will not be possible to determine a reliable degree of functional conservation of the different Wnt ligands.

There is also an imbalance in the analysis of the different *wnt* genes, mainly concentrated in the posterior expression and function of Wnt1 and Wnt8 ligands. This analysis would be greatly benefited by incorporating the complete set of Wnt ligands in more organisms, including representatives of orders not analyzed so far (*e.g.*, hemimetabolous insects, non-arachnid chelicerates).

## Functional analysis of Wnt ligands during posterior growth

One of the main characteristics of panarthropods is their segmented body, the segments of which are added during development in different ways depending on the species. For example, in *Drosophila*, all segments are patterned almost simultaneously during syncytial blastoderm in what is called long germ segmentation (Peel et al., 2005). On the other hand, most arthropods and onychophorans present a short germ mode of segmentation, where anterior segments are patterned during the blastoderm stage, and the rest of segments are added sequentially from the SAZ, at the posterior end of the embryo/larva (Peel et al., 2005; Clark and Peel, 2018). This territory functions as an organizing center for posterior growth, where the mechanisms that regulate segmentation and axial elongation occur (Williams and Nagy, 2017). As was mentioned previously, the SAZ is characterized by expressing several Wnt ligands dynamically during segmentation, suggesting a role of this pathway in establishing the segmented pattern and the mechanisms controlling body elongation.

Functional studies on different components of the Wnt pathway during sequential segmentation have been performed mostly in the holometabolous insect *Tribolium castaneum* (Ober and Jockusch, 2006; Bolognesi et al., 2008b; Bolognesi et al., 2009; Beermann et al., 2011) and the spider *Parasteatoda tepidariorum* (McGregor et al., 2008; Setton and Sharma 2018; Setton and Sharma 2021), although the number of studies in other arthropod species is increasing.

**FIGURE 4:**
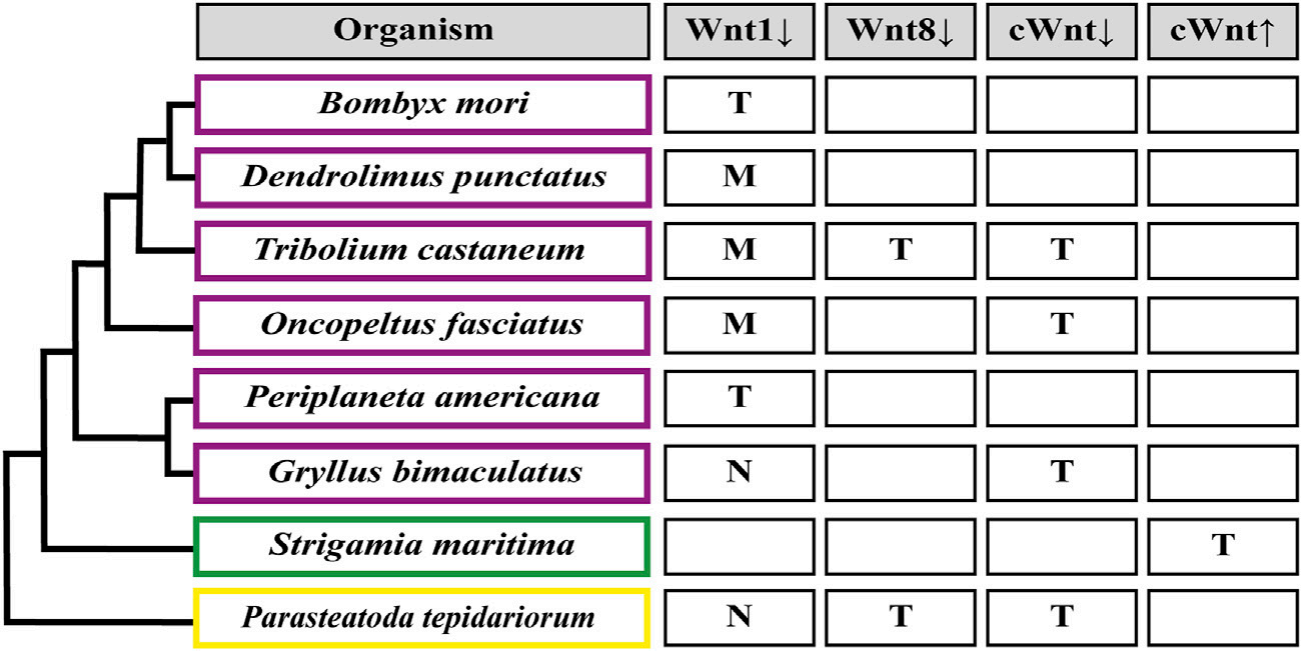
Segmentation patterning phenotypes after Wnt signaling functional analysis. (M) Loss of segment boundaries. (T) Truncated embryo due to loss of abdominal segments. (N) No effect on the segmentation patterning. Down arrow indicates inhibition, up arrow indicates activation. cWnt indicates the full canonical pathway. An empty rectangle points out the absence of the corresponding functional studies in this organism. Insects are shown in purple rectangles (Miyawaki et al., 2004; Angelini and Kaufman, 2005; Bolognesi et al., 2008b; Chesebro et al., 2013; Liu et al., 2017; Nakao, 2018; Setton and Sharma, 2021); myriapods in green (Hayden et al., 2015); chelicerates in yellow (McGregor et al., 2008; Setton and Sharma, 2021).

Mainly, it has been observed that the loss of function of Wnt receptors in *Tribolium* (*Tc-frizzled1* and *Tc-frizzled2*), co-receptor Arrow in *Tribolium*, *Oncopeltus*, *Gryllus* and *Parasteatoda* (*Tc-arrow*, *Of-arrow*, *Gb-arrow* and *Pt-arrow*, respectively), or final effectors (the transcription factors *Gb-armadillo/β-catenin* in *Gryllus* and *Of-pangolin* in *Oncopeltus*) of the canonical Wnt pathway, lead to the formation of truncated embryos with no posterior segments (Figure 4) (Miyawaki et al., 2004; Angelini and Kaufman 2005; Bolognesi et al., 2009; Beermann et al., 2011; Setton and Sharma, 2018; Setton and Sharma, 2021). This makes clear the essential role of this signaling pathway on the posterior growth. The same conclusion could be reached by analyzing the pharmacological inhibition of the Wnt pathway after the incubation of elongating cockroach embryos with the Inhibitor of Wnt Production-3 (IWP-3)—that blocks palmitoylation of Wnt ligands by Porcupine (Chen et al., 2009)—and after Wnt signaling activation using LiCl–that inhibits GSK3 within the *β*-catenin destruction complex (Hedgepeth et al., 1997)—during the elongation and segmentation of the centipede *Strigamia maritima*. After the IWP-3 treatment, the absence of Wnt signaling specifically during elongation disrupted segment formation and reduced the size of the SAZ as well as the number of proliferating cells, coinciding with the results showed by Oberhofer et al. (2014) where the Wnt signaling displays a dual role in the SAZ in growth control and posterior patterning. In the latter, the activation of the pathway generated shorter embryos with an expanded SAZ, revealing its role in the control of the segmentation clock (Chesebro et al., 2013; Hayden et al., 2015).

Regarding Wnt ligands, by far the most studied is *wnt1*/*wingless*, but its functional analysis has only been successful in some insects, with no loss-of-function phenotypes found in the cricket *G. bimaculatus* and the spider *P. tepidariorum* (Miyawaki et al., 2004; Setton and Sharma, 2021). Apparently, the severity of *wnt1* knockdown related to posterior growth could be linked to the presence/absence of another ligand, *wnt8*: In *Tribolium* and *Oncopeltus*–where Wnt8 ligand is present as one and two paralogs, respectively–*wnt1* RNAi only showed segmental boundary disruption without preventing elongation or the generation of the segmented pattern, as was revealed by the absence of truncated phenotypes and the regular expression of the pair-rule gene *Tc-even-skipped* (*Tc-eve*) in the beetle and a normal-sized abdomen in the case of *Oncopeltus* embryos (Angelini and Kaufman, 2005; Ober and Jockusch, 2006; Bolognesi et al., 2008b). On the other hand, in the domestic silk moth *Bombyx mori*, which do not have a *wnt8* paralog, *wnt1* RNAi produced a range of phenotypes from partially truncated embryos displaying immature legs (Yamaguchi et al., 2011) to drastically shortened embryos showing no signs of abdominal nor thoracic segments (Nakao, 2018). The idea of a (partial) functional interrelationship between both ligands is also based on the results obtained in *Tribolium* after eliminating *wnt8* alone, that generated a small proportion of truncated embryos, and the drastic increasing of the truncated phenotype frequency using the *wnt1* and *wnt8* double RNAi (Bolognesi et al., 2008b). There are other cases in which the same different results have been reported: In the cockroach *Periplaneta americana*, the loss-of-function of *wnt1* led embryos to develop posteriorly truncated germbands (Chesebro et al., 2013), while in the lepidopteran *Dendrolimus punctatus*, *wnt1* knockout using CRISPR/Cas9 showed abdominal segment fusion with no signs of posterior truncation (Liu et al., 2017). However, it is not known if Wnt8 ligand is present in these two insects. Finally, *wnt8* knockdown in the spider *Parasteatoda* (McGregor et al., 2008) caused posterior truncation or even complete absence of the opisthosomal (abdominal) region, a phenotype that resembles the effect of the RNAi against *wnt8* in *Tribolium* (Bolognesi et al., 2008b). However, given the functional redundancy between Wnt1 and Wnt8 ligands, we cannot interpret *wnt1* or *wnt8* knockdown phenotypes in any species where *wnt1*/*wnt8* double loss-of-function has not been analyzed.

The analysis of the phenotypes mentioned above reveals the Wnt pathway’s different roles during posterior growth. On the one hand, truncated phenotypes indicate early participation in this process, although we have little evidence about the mechanisms in which it would operate. In turn, we do not have sufficient evidence to relate the Wnt signaling to maintaining gene oscillations or establishing a wavefront at the anterior SAZ, as it is well known in vertebrates (Oates et al., 2012). On the other hand, the participation of *wnt1* in the definition of segment borders is relatively conserved among arthropods and is a well-understood process at the molecular and cellular level in the *Drosophila* fly (Nüsslein-Volhard and Wieschaus, 1980; Nagy and Carroll 1994; Ober and Jockusch, 2006).

To date, other canonical and noncanonical Wnt ligands that are expressed segmentally and/or in the posterior region have only been functionally studied in the beetle *Tribolium*. When Bolognesi et al. (2008b) RNAi screened all nine Wnt ligands expressed in *Tribolium*, they only found segmental phenotypes with *Tc-wnt1* and *Tc-wnt8*. The same result was obtained after checking different combinations of double (all combinations of *Tc-wnt1*, *Tc-wnt5*, *Tc-wnt8* and *Tc-wntA*) and triple (*Tc-wnt5*, *Tc-wnt8* and *Tc-wntA*) RNAi injections of the *wnt* genes that are most clearly expressed in the SAZ (Bolognesi et al., 2008a, b). However, we cannot rule out redundant functions during posterior growth for *wnt5* and *wntA* with *wnt6*, which is also expressed in the SAZ but was not included in their double and triple knockdown analyses.

It is also possible that in other panarthropods, different *wnt* genes fulfill the absence of specific Wnt ligands, like Wnt8 in *Thamnocephalus* (Constantinou et al., 2016), *Strigamia* (Hayden et al., 2015) or the onychophoran *Euperipatoides* (Hogvall et al., 2014). The evidence suggests redundant and partially redundant functions between arthropod Wnt ligands. Nevertheless, the relationship between a Wnt ligand and its precise function and which ligands share the same function is still unclear.

## Wnt signaling as part of the genetic network underlying segmental patterning

It has been established that segmentation in most arthropods and all vertebrates is driven by a cyclical mechanism where the temporal periodicity of a clock is translated into a repetitive spatial pattern (Cooke and Zeeman, 1976; Palmeirim et al., 1997; Sarrazin et al., 2012). In vertebrates, cell-autonomous oscillatory genes expression is maintained by a posterior Wnt + Fgf signaling gradient. As this gradient decreases, the generated posterior-to-anterior kinematic waves of expression are arrested, allowing the formation of a new segment (Oates et al., 2012). Furthermore, the progressive posterior elongation of the embryo moves the Wnt + Fgf gradient together with the determination front, thus linking posterior growth with segmentation. In addition, several genes related to the Wnt, Fgf, and Notch/Delta pathways oscillate and are coupled between them, which is crucial for segment generation (Dequéant et al., 2006; Krol et al., 2011; Sonnen et al., 2018). However, despite the evidence involving the Wnt signaling pathway in the vertebrate segmentation clock, its specific role is not yet fully understood.

In arthropods, *caudal*, a highly conserved gene that is expressed at the posterior half of the SAZ and is crucial for its establishment and maintenance (Dearden and Akam, 2001; Chipman et al., 2004; Copf et al., 2004; Shinmyo et al., 2005; Chesebro et al., 2013; Janssen et al., 2015; Schönauer et al., 2016; Novikova et al., 2020), is regulated by the canonical Wnt signaling (Shinmyo et al., 2005; McGregor et al., 2008; Chesebro et al., 2013; Oberhofer et al., 2014; Schönauer et al., 2016). Recently, Clark and Peel (2018) proposed that in the SAZ, Wnt signaling would be regulating an ancient regulatory network formed by *caudal*, *dichaete*, and *odd-paired* (*opa*), which are sequentially expressed–both temporally and spatially–during segmentation in *Tribolium*, but also in the long-germ *Drosophila* embryo. Furthermore, one of the same authors proposed a transition from the posterior expression of *caudal*/*dichaete* to *dichaete*/*opa* expression at the anterior region of the SAZ, resembling the wavefront limit operating in vertebrates (Clark, 2021). Then it is possible that the Wnt pathway is regulating, through Caudal, genetic oscillations in arthropods, as occurs in vertebrates.

Two main kinds of cyclically expressed genes have been observed in arthropods (no dynamically expressed genes have been found in onychophorans; Janssen and Budd, 2013; Janssen and Budd, 2016): On one hand, the orthologs of *Drosophila* pair-rule genes as *odd-skipped* (Chipman et al., 2004; Sarrazin et al., 2012), *even-skipped* (Chipman and Akam, 2008; El-Sherif et al., 2012; Brena and Akam, 2013), *paired* (Eriksson et al., 2013), *hairy* (Chipman and Akam, 2008; Pueyo et al., 2008) and most probably *runt* (Schönauer et al., 2016). On the other hand, members of the Notch signaling pathway, mainly the Delta ligand, have been shown to oscillate in cockroaches (Pueyo et al., 2008), centipedes (Chipman and Akam, 2008; Brena and Akam, 2013) and spiders (Stollewerk et al., 2003; Schönauer et al., 2016). Functional analyses in a few of these organisms appear to position Wnt signaling upstream of some cyclic genes.

In *Tribolium*, it was already shown that blocking Wnt signaling impairs the posterior expression of the three pair-rule genes (*Tc-run*, *Tc-eve*, and *Tc-odd*; Bolognesi et al., 2009; Beermann et al., 2011), which are part of the regulatory circuit involved in the segmentation clock (Choe et al., 2006). In the same insect, RNA-seq comparative analysis revealed additional pair-rule genes downregulated after knocking down the Wnt/β-catenin pathway, such as *Tc-hairy* and *Tc-dichaete*, in addition to *Tc-odd* and *Tc-eve* (Oberhofer et al., 2014). However, the same experimental approach performed in the spider *Parasteatoda* showed no significant effect on the expression of the pair-rule genes *Pt-eve*, *Pt-run*, and *Pt-hairy*, with a mild effect on *Pt-dichaete* (Setton and Sharma, 2021). In this study, it was even seen that *caudal* expression was not interrupted in *Parasteatoda* and *Gryllus* after arrow knockdown, unlike in *Oncopeltus*, where they obtained a substantial reduction in *Of-caudal* expression. The discrepancies observed between *Tribolium* and *Parasteatoda* Wnt/β-catenin pathway loss-of-function results could be associated with the weak penetrance of the RNAi injection (around 50% of effect; Setton and Sharma, 2021) or with a possible low conservation of the *wnt*/*caudal*/pair-rule gene network among arthropods. In any case, if we consider that in the spider *Parasteatoda wnt8* was previously shown to be necessary for the posterior expression of *Pt-eve* and *Pt-run* (Schönauer et al., 2016) and *Pt-caudal* (McGregor et al., 2008; Schönauer et al., 2016), one alternative explanation could be that specifically in the spider, Wnt8 function acts through an Arrow-independent pathway, that is like a noncanonical ligand.

Regarding Wnt-Notch interactions, on the other hand, Chesebro et al. (2013) showed in the cockroach that *Pa-delta* expression in the posterior SAZ is completely eliminated after *Pa-wnt1* knockdown. Furthermore, they obtained similar results using the pharmacological inhibitor IWP-3 (Chesebro et al., 2013). At the same time, they observed the disruption of posterior *Pa-wnt1* expression after injecting RNAi against *Pa-notch* and its partial reduction by incubating embryos with the Notch inhibitor, DAPT.

Schönauer et al. (2016), in turn, situated this signaling pathway upstream *Pt-wnt8* in *Parasteatoda*. Depending on the SAZ region, Notch/Delta is activating (at the posterior SAZ) or inhibiting (at the anterior SAZ) this ligand. However, it was previously found that Wnt8 would be necessary for the dynamic expression of *Pt-delta* in the same spider (McGregor et al., 2008). In addition, the RNA-seq study by Setton and Sharma (2021) exhibited *Pt-notch* ″downregulation-but not Pt delta inhibition-after″ Wnt signaling loss-of-function, although they did not check the effect of knocking down Notch signaling. So then, Notch and Wnt signaling pathways seem to display a reciprocal regulation in these two animal models, the hemimetabolous cockroach *Periplaneta* and the chelicerate *Parasteatoda*, apparently partially through their regulation of *caudal*. Nevertheless, our limited knowledge about the complexity of the SAZ in terms of cell types and tissue organization makes it hard to understand reciprocal interactions between different pathways.

**FIGURE 5:**
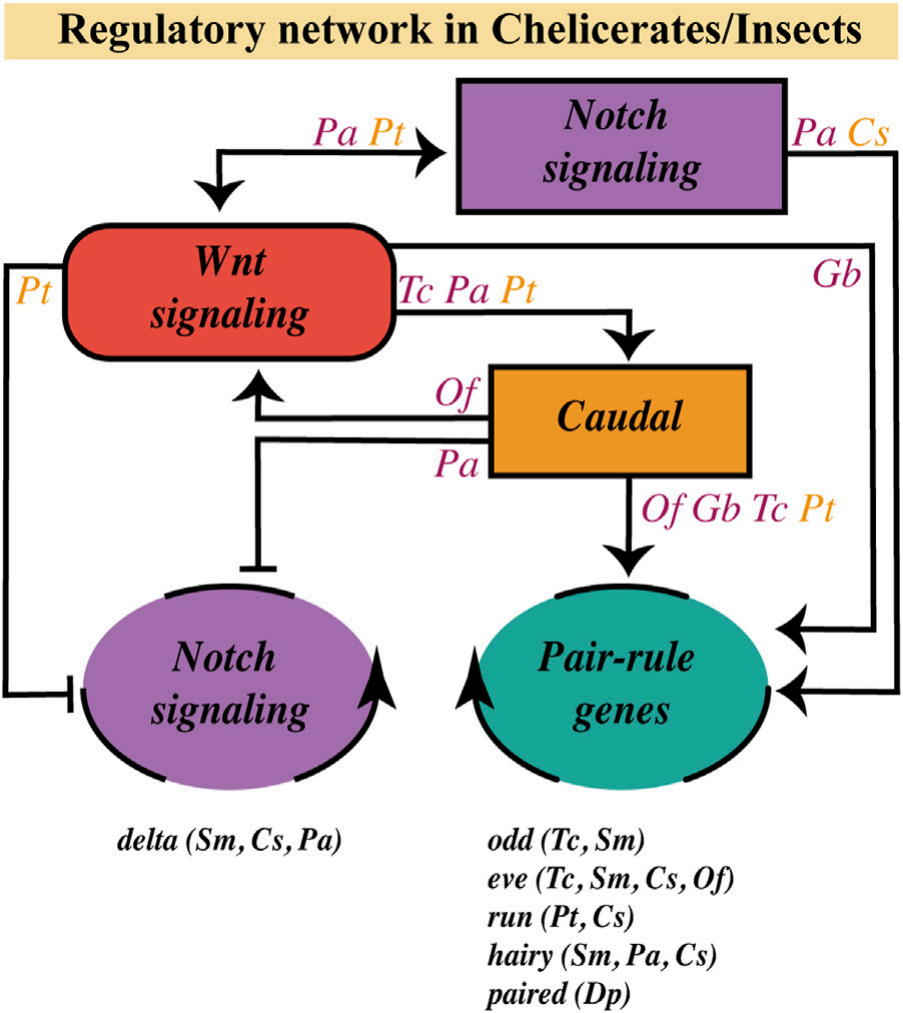
Wnt pathway role on the segmentation gene network at the SAZ. Regulatory relationships of the Wnt signaling pathway with the Notch pathway, the *caudal* gene and the dynamic/oscillatory genes (belonging to the Notch pathway or to the Pair-Rule gene family) in the SAZ, based on current evidence in chelicerates (orange letters) and insects (purple letters). Genes with a dynamic/oscillatory behavior at the SAZ in different arthropods are shown at the bottom. *C. salei* (*Cs*); *D. pulex* (*Dp*); *G. bimaculatus* (*Gb*); *O. fasciatus* (*Of*); *P. americana* (*Pa*); *P. tepidariorum* (*Pt*); *S. maritima* (*Sm*); *T. castaneum* (*Tc*).

As it has been suggested that the Notch pathway does not present a functional role in the segmentation process in the onychophoran *Euperipatoides kanangrensis* and the holometabolous insects *Drosophila melanogaster, Apis mellifera* and *Tribolium castaneum* (Aranda et al., 2008; Wilson et al., 2010; Kux et al., 2013; Janssen and Budd, 2016), the regulatory role of the Wnt pathway on the Notch signaling is limited, considering the existing evidence, to *Parasteatoda tepidariorum*, *Periplaneta americana* and the short-germ silkworm *Bombyx mori* (Liu, 2013). Thus, from the cases mentioned so far, we can conclude the following (Figure 5):

1) Studies in different arthropods are lacking to determine a possible ancestral regulatory route of posterior growth.
2) Virtually all arthropods studied to date have shown some level of dynamic/oscillatory expression in the SAZ (*i.e.*, *odd*, *eve*, *run*, *hairy*, *paired*, *delta*), and the most widely described regulatory network involved is Wnt > Caudal > Pair-Rule/Delta - with some variations possible due to evolutionary divergences or even experimental limitations -, where Wnt signaling has a regulatory effect on the timing of segment border definition.
3) Notch signaling, as in vertebrates, could be involved in single-cell oscillations coupling in some arthropods and not others, where we have not yet discovered the gene(s) involved in the oscillatory synchronization.

## Wnt pathway as a possible regulator of the cellular processes involved in axial elongation?

Sequential segment addition in most arthropods is accompanied by simultaneous germband extension along the anterior-posterior axis. This complex reorganization of the developing body must include, in a species-specific manner, a variable, mutually dependent, and tightly regulated arrangement of cellular behaviors, including oriented (or not) mitotic divisions, cell rearrangements, and changes in cellular density, cell shape, and cell size, among others.

Little is known about the regulatory network behind axial elongation and the mentioned cellular processes in arthropod posterior growth. In deuterostomes, including vertebrates as well as the hemichordates that display posterior growth without segmentation, paraxial mesoderm progenitors are maintained by a posterior positive regulatory loop between *wnt* (*wnt3a* and *wnt8* in zebrafish) and *brachyury*, so that posterior elongation depends on this molecular feedback (Martin and Kimelman, 2008; Fritzenwanker et al., 2019). However, *brachyury* orthologs in insects are expressed in the SAZ but seem not necessary for posterior growth, as was shown by RNAi analysis in *Tribolium* and *Gryllus* (Shinmyo et al., 2006; Berns et al., 2008). Regardless of whether Wnt-Brachyury regulation was lost in insects or corresponds to a deuterostome evolutionary innovation, which factor is maintaining Wnt signaling at the insect SAZ is unknown.

Few direct evidence links Wnt signaling to cell proliferation during arthropod posterior growth; for example, Oberhofer et al. (2014), through genetic analyses in *Tribolium* SAZ, showed that the Wnt pathway targets some genes involved in mitotic cell cycle and spindle organization/elongation. Moreover, RNAi treatments against *wnt1* caused a decrease in cell proliferation in the cockroach SAZ, the same occurring when the pathway was inhibited by pharmacological treatment with IWP-3 (Chesebro et al., 2013). Constantinou et al. (2020), based on the cell division pattern shown by the branchiopod *Thamnocephalus*, divided the SAZ into an anterior no-proliferating region that expresses WntA ligand and a posterior slightly-but-constant proliferating region that expresses Wnt4 ligand. A similar correlation was found in *Oncopeltus* (Auman et al., 2017), where the region of cell divisions coincides with the expression of *Of-eve* and *Of-caudal* genes, two targets of Wnt signaling. The proposed model differs, however, from what was found in *Dermestes* (Xiang et al., 2017) and *Tribolium* (Cepeda et al., 2021). The proliferation region is not temporally stable in these beetles, but variable and posteriorly concentrated at specific stages. Unfortunately, no correlation has yet been found between this proliferation pattern and the expression of any gene at the SAZ, and there is evidence that the Wnt signaling target genes *Tc-caudal*, and *Tc-eve* are not involved in cell division regulation in this insect (Copf et al., 2004; Nakamoto et al., 2015). A possible mechanism that explains this patterning type would be what happens in the *Drosophila* wing, where cell proliferation is regulated by differences in the amount of Wnt1 ligand; high concentrations generate a decrease while intermediate concentrations trigger proliferation (Baena-Lopez et al., 2009). However, no changes in the expression of Wnt ligands correlating with this model have been demonstrated.

Another well-conserved cellular process among arthropods during germband elongation is the convergent extension (CE). The first studies on CE were carried out in *Drosophila*, where the pair-rule genes *Dm-eve* and *Dm-runt* are necessary for the polarized cell movements involved (Irvine and Wieschaus, 1994; Zallen and Wieschaus, 2004). Furthermore, the necessity of *Tc-eve* and *Tc-caudal* during the intercalary cell movements seen at early stages of germband elongation in *Tribolium* was also reported (Benton et al., 2013; Nakamoto et al., 2015). In addition, the fact that *Dm-eve* regulates the expression of the Toll family receptors, which direct CE during germband elongation in *Drosophila* (Paré et al., 2014) and is conserved in *Tribolium* and *Parasteatoda* (Benton et al., 2016), leads us to wonder if the Wnt pathway would have a role in this process. The Wnt signaling pathway could be involved in CE through its regulation of *eve* expression, which we have already seen occur in various arthropods during germband elongation (Bolognesi et al., 2009; Beermann et al., 2011; Oberhofer et al., 2014; Schönauer et al., 2016).

However, we will not have an answer to our question until a more significant number of studies have been carried out with a particular focus on the cellular behaviors that take place during posterior elongation in a diverse group of arthropods.

## Future directions of research

After reviewing the Wnt pathway’s role in axial elongation and segmentation processes in arthropods, more questions than answers arise.

We found the complete Wnt ligand repertoire from 42 species of panarthropods. However, the expression pattern of all the ligands has been published only in twelve. Thus, a more diverse palette of species is lacking in the analysis, especially non/underrepresented lineages (*e.g.*, maxillopod crustaceans, non-insect hexapods, non-spider chelicerates). Furthermore, given the apparent overlapping between some ligands, it would be precious to have a detailed cellular-level analysis of the expression patterns, hopefully including double *in situ* hybridizations.

The incorporation of functional studies in more organisms, including non-model panarthropods with interesting phylogenetic positions as early-branching clades (*e.g.*, onychophorans, ametabolous and hemimetabolous insects), will provide the basis for well-supported comparative analyses. In the long term, this will allow us to identify conserved and diverged aspects of the regulatory role of the Wnt pathway on posterior body elongation and segmentation. Furthermore, adding imaging tools to this analysis, like tissue-specific transgenic lines carrying fluorescent reporters for time-lapse imaging, will be essential to understanding the contribution of Wnt signaling in the highly dynamic cellular and molecular network that organizes posterior growth in most panarthropods.
